# Clinical impact of CEUS on non-characterizable observations and observations with intermediate probability of malignancy on CT/MRI in patients at risk for HCC

**DOI:** 10.1007/s00261-024-04305-9

**Published:** 2024-06-11

**Authors:** Yuko Kono, F. Piscaglia, S. R. Wilson, A. Medellin, S. K. Rodgers, V. Planz, A. Kamaya, D. T. Fetzer, A. Berzigotti, P. S. Sidhu, C. E. Wessner, K. Bradigan, Cristina M. Kuon Yeng Escalante, T. Siu Xiao, J. R. Eisenbrey, F. Forsberg, A. Lyshchik, CEUS LI-RADS Trial Group, Gibran T. Yusuf, Abid Suddle, Vasileios D. Rafailidis, Lorenzo Mulazzani, Alessandro Granito, Eleonora Terzi, Antonella Forgione, Alice Giamperoli, Bernardo Stefanini, Iuliana-Pompilia Radu, Lisa Finch, Amit G. Singal

**Affiliations:** 1 Division of Gastroenterology and Hepatology, Department of Medicine, University of California, San Diego, La Jolla, CA USA; 2 Division of Internal Medicine, Hepatobiliary and Immunoallergic Diseases, IRCCS Azienda Ospedaliero-Universitaria di Bologna, Bologna, Italy; 3 Department of Medical and Surgical Sciences, University of Bologna, Bologna, Italy; 4 University of Calgary, Calgary, Canada; 5 Thomas Jefferson University Hospital, Philadelphia, PA USA; 6 Einstein Medical Center, Philadelphia, PA USA; 7 Vanderbilt University, Nashville, TN USA; 8 Stanford University, Stanford, CA USA; 9 UT Southwestern Medical Center, Dallas, TX USA; 10 Department of Visceral Surgery and Medicine, Bern University Hospital, University of Bern, Bern, Switzerland; 11 Department of Imaging Sciences, School of Biomedical Engineering and Imaging Sciences, Faculty of Life Sciences and Medicine, King’s College London, London, UK; 12 Department of Radiology, King’s College Hospital, London, UK

**Keywords:** Hepatocellular carcinoma, Diagnosis, CEUS, LI-RADS

## Abstract

**Background:**

Hepatocellular carcinoma (HCC) is a unique cancer allowing tumor diagnosis with identification of definitive patterns of enhancement on contrast-enhanced imaging, avoiding invasive biopsy. However, it is still unclear to what extent Contrast-Enhanced Ultrasound (CEUS) is a clinically useful additional step when Computed tomography (CT) or Magnetic resonance imaging (MRI) are inconclusive.

**Methods:**

A prospective international multicenter validation study for CEUS Liver Imaging Reporting and Data System (LI-RADS) was conducted between January 2018 and August 2021. 646 patients at risk for HCC with focal liver lesions were enrolled. CEUS was performed using an intravenous ultrasound contrast agent within 4 weeks of CT/MRI. Liver nodules were categorized based on LI-RADS (LR) criteria. Histology or one-year follow-up CT/MRI imaging results were used as the reference standard. The diagnostic performance of CEUS was evaluated for inconclusive CT/MRI scan in two scenarios for which the AASLD recommends repeat imaging or imaging follow-up: observations deemed non-characterizable (LR-NC) or with indeterminate probability of malignancy (LR-3).

**Results:**

75 observations on CT or MRI were categorized as LR-3 (*n* = 54) or LR-NC (*n* = 21) CEUS recategorization of such observations into a different LR category (namely, into one among LR-1, LR-2, LR-5, LR-M, or LR-TIV) resulted in management recommendation changes in 33.3% (25/75) and in all but one (96.0%, 24/25) observation, the new management recommendations were correct.

**Conclusion:**

CEUS LI-RADS resulted in management recommendations change in substantial number of liver observations with initial indeterminate CT/MRI characterization, identifying both non-malignant lesions and HCC, potentially accelerating the diagnostic process and alleviating the need for biopsy or follow-up imaging.

ClinicalTrials.gov number, NCT03318380.

## Introduction

Hepatocellular carcinoma (HCC) is among the most lethal cancers worldwide, though with improved survival when accurately diagnosed at an early, curative stage [1]. Compliance with HCC surveillance programs help accomplish this goal [2]. Unlike most solid cancers, HCC diagnosis and treatment planning can often be confidently established through noninvasive dynamic contrast imaging without the need for biopsy. Consequently, the precision of imaging diagnosis is of paramount importance. To ensure a standardized approach to the technique, terminology, interpretation, and reporting of liver imaging in individuals at risk for HCC, the Liver Imaging Reporting and Data System (LI-RADS) was established. Initially developed in 2011 for computed tomography (CT) and magnetic resonance imaging (MRI), LI-RADS was expanded to include contrast-enhanced ultrasound (CEUS) in 2017 [3].

The current American Association for the Study of Liver Diseases (AASLD) practice guidance on the prevention, diagnosis, and treatment of HCC recommends US as the first-line imaging modality for HCC surveillance, and contrast-enhanced, multiphase CT and MRI for diagnosis of HCC in patients with nodules ≥ 1 cm detected by surveillance [2]. These guidelines also accept the possibility of CEUS as a second-line modality in cases where MRI and CT are inconclusive, unavailable, or contraindicated, or when tumor biopsy is not feasible [2]. The National Comprehensive Cancer Network (NCCN) Guidelines for Hepatobiliary Cancers do not include CEUS in the HCC diagnosis algorithm as of this publication, though mention that CEUS has comparable diagnostic accuracy as MRI, albeit not commonly used in the United States of America (USA) [4]. Practice guidelines from international organizations such as the European Association for the Study of the Liver (EASL) [5], Asian Pacific [6], Canadian [7], Korean [8], and Japanese [9] HCC guidelines include CEUS in their respective diagnostic algorithms as a second-line modality. Notable strengths of CEUS include resilience to mistiming of the arterial phase (common occurrence with CT/MRI), ability to administer multiple doses of contrast agent, capability to detect subtle enhancement differences, and a favorable contrast agent safety profile [10].

Our group recently conducted an international multicenter prospective validation study of CEUS LI-RADS in patients at risk for HCC in North America and Europe [11]. The study demonstrated that CEUS LI-RADS (LR) accurately categorizes liver nodules in participants at risk for HCC, achieving a 95.1% specificity and 97.0% positive predictive value (PPV) of LR-5 for HCC diagnosis. In that study and as well as in our daily clinical practice, a substantial number of liver observations in patients at risk for HCC are categorized on CT/MRI as LR-3 (indeterminate probability for malignancy). There are also cases categorized as LR-NC (non-characterizable). This categorization is assigned when observations cannot be categorized meaningfully because key imaging phases were omitted or degraded, preventing assessment of one or more major features. As a direct result, reasonable categories range from those where cancer is unlikely (LR-1 or LR-2) to those where cancer is likely (LR-4, LR-5, LR-M). According to the AASLD guidelines, observations with both LR-3 and LR-NC categorization are managed by repeat or alternating CT/MRI imaging, but such an approach may delay HCC diagnosis, bearing the risk of leaving an active aggressive cancer untreated. Interestingly, HCCs without a typical conclusive imaging contrast pattern are not less aggressive than those with the typical diagnostic features [12].

We hypothesize that CEUS is capable of re-categorizing CT/MRI-indeterminate lesions with high accuracy, resulting in meaningful management recommendation changes. We therefore conduct a sub-analysis to explore the clinical impact of CEUS to further characterize focal liver nodules with LR-3 and LR-NC CT/MRI categorization.

## Methods

A prospective international multicenter validation study for CEUS LI-RADS was conducted between January 2018 and August 2021 [11]. This study was reviewed and approved by the Institutional Review Board at each participating institution. Before enrollment, written informed consent was obtained from all participants. The study was performed at 11 academic and nonacademic sites in the USA, Canada, Italy, the UK, and Switzerland. Extensive clinical site monitoring and data auditing were conducted to ensure that the rights of all study participants were protected, the study was implemented in accordance with the protocol, and the data collection methods and the quality and integrity of study data were maintained.

The subject population included patients at risk for HCC presenting with untreated focal liver nodules detected on standard-of-care screening US or observation on diagnostic CT or MRI. A total of 646 consecutive patients at risk for HCC were enrolled in this study. The study inclusion and exclusion criteria were previously reported [11].

CEUS was performed using the intravenous US contrast agent Sulfur hexafluoride lipid type A microspheres (Lumason/SonoVue, Bracco Diagnostic) within 4 weeks of multiphase contrast-enhanced CT/MRI or 4 weeks before tissue sampling. We performed a urine pregnancy test in women of childbearing age before each CEUS study, the results of which were made available to the subject prior to study initiation.

All liver observations were categorized based on CEUS and CT/MRI LI-RADS 2017 criteria. CEUS LI-RADS categorization was performed by a physician either performing or supervising CEUS examinations at each participating institution [6]. Similar to routine clinical practice, the readers of CEUS were not purposefully blinded to the results of CT/MRI, since CT/MRI results commonly used to guide CEUS examinations.

All centers involved in this study had personnel familiar with CEUS and with appropriate knowledge and technical skills. All sites had access to CEUS educational materials and on-site clinical application support to ensure appropriate use of ultrasound contrast agent and ultrasound scanners. In addition, all study personnel had access to CEUS educational materials on technical recommendations, APHE and washout assessment, and a copy of pictorial essay recently created by the ACR CEUS LI-RADS working group that provides imaging examples. CEUS examiners had between 3 and 25 years of experience in liver CEUS. The LI-RADS categories were assigned as follows: non-characterizable observations (LR-NC); Tumor in Vein (LR-TIV); LR-1 or LR-2 as benign or probably benign nodules, respectively; CEUS LR-M as probably or definitely malignant but not HCC specific; and all other nodules assigned categories of LR-3 (intermediate probability of malignancy), LR-4 (probably HCC), or LR-5 (definitely HCC) according to CEUS LI-RADS [6].

The present analysis focused on the clinical utility of CEUS in observations that were categorized as LR-3 or LR-NC on CT or MRI and therefore require imaging follow-up or alternative imaging modality, according to the recent AASLD guidelines.

For the purpose of this study, all management decisions were made solely on CT/MRI or tissue histology results.

All CT and MRI examinations were interpreted by site radiologists according to CT/MRI LI-RADS v 2018.

The composite reference standard to categorize observations as “HCC,” “Non-HCC malignancy,” or “non-malignant” was based on histopathology, initial or follow-up CT, or MR imaging in the following order of strength as previously reported [11]:

1. Histopathology (biopsy, surgical excision, or explant histology) was used whenever available for all observations, regardless of their initial imaging characterization.
2. For observations without histopathology, imaging follow-up was performed for up to 1 year from the initial examination. Results of follow-up imaging were used as follows:
  - Observations downgraded to CT/MRI LR-1 or LR-2 on 1-year follow-up imaging were considered non-malignant.
  - Observations with CT/MRI LR-3 categorization on 1-year follow-up imaging were considered non-malignant.
  - Observations with CT/MRI LR-4 categorization on 1-year follow-up imaging were excluded from the analysis due to the uncertainty of imaging diagnosis.
  - Observations upgraded to CT/MRI LR-5 on any follow-up imaging were considered HCC.
  - Observations upgraded to CT/MRI LR-M or CT/MRI LR-TIV on any follow-up imaging in patients without contraindications to biopsy were referred for histological confirmation, as per current clinical practice standards. CT/MRI LR-M and CT/MRI LR-TIV lesions without histological confirmation were excluded from the analysis.

### Statistical methods

To assess the clinical impact of CEUS in patients with CT/MRI LR-3 and LR-NC categorization, we calculated the proportion of patients in which CEUS resulted in changes of management recommendations as defined by the AASLD Clinical Guidance on HCC version 2023. We also calculated positive predictive value (PPV) and negative predictive value (NPV) of CEUS diagnosis. All statistical analyses were performed using Statistical Analysis Software (SAS) 9.4 (SAS Institute, Cary, NC, USA).

## Results

There were a total of 75 nodules in 68 patients categorized as LR-3 (72%, 54/75) or LR-NC (28%, 21/75) on CT/MRI. Patient demographics and relevant clinical information are shown in Table 1.

**Table 1:** Patient’s characteristics

	Total	Percentages
Gender		
Female	19	27.9
Male	49	72.1
Race		
American Indian or Alaska Native	0	0
Asian	6	8.8
African American	7	10.3
Native Hawaiian or Other Pacific Islander	1	1.5
White	48	70.5
Other	5	7.4
Unknown	1	1.5
Ethnicity		
Hispanic or Latino	6	8.9
Not Hispanic or Latino	60	88.2
Unknown	2	2.9
Personal history of HCC		
Yes	16	23.5%
No	52	76.5%
Personal history of treated HCC		
Yes	13	19.1%
No	55	80.9%
Liver disease etiology		
Alcohol	24	29.2%
NASH	15	18.3%
Hepatitis B	9	11.0%
Hepatitis C	25	30.5%
Other	9	11.0%
Cirrhosis		
Yes	66	97.1%
No	2	2.9%
Cirrhosis biopsy proven		
Yes	5	7.4%
No	61	89.7%
Unknown	2	2.9%
Encephalopathy		
No encephalopathy	62	91.2%
Grade 1–2	3	4.4%
Grade 3–4	0	0.0%
Unknown	3	4.4%
Ascites		
Absent	43	63.3%
Slight	20	29.4%
Moderate	2	2.9%
Unknown	3	4.4%
ECOG performance status		
0	52	76.5%
1	5	7.4%
2	4	5.9%
3	1	1.5%
4	0	0.0%
5	0	0.0%
Unknown	6	8.9%
Child–Pugh score		
A	43	63.3%
B	21	30.9%
C	2	2.9%
Unknown	2	2.9%

### Observations categorized as LR-3 on CT/MRI

There were a total of 54 observations categorized as LR-3 on CT or MRI, of which 25.9% (14/54) were confirmed as HCC, 72.2% (39/54) were non-malignant, and 1.9% (1/54) non-hepatocellular malignancy (mixed hepatocellular cholangiocarcinoma). The final diagnosis reference standard was based on follow-up CT/MRI (74.1%, 40/54), percutaneous biopsy (14.8%, 8/54), or liver explant histology (11.1%, 6/54).

**Fig. 1:**
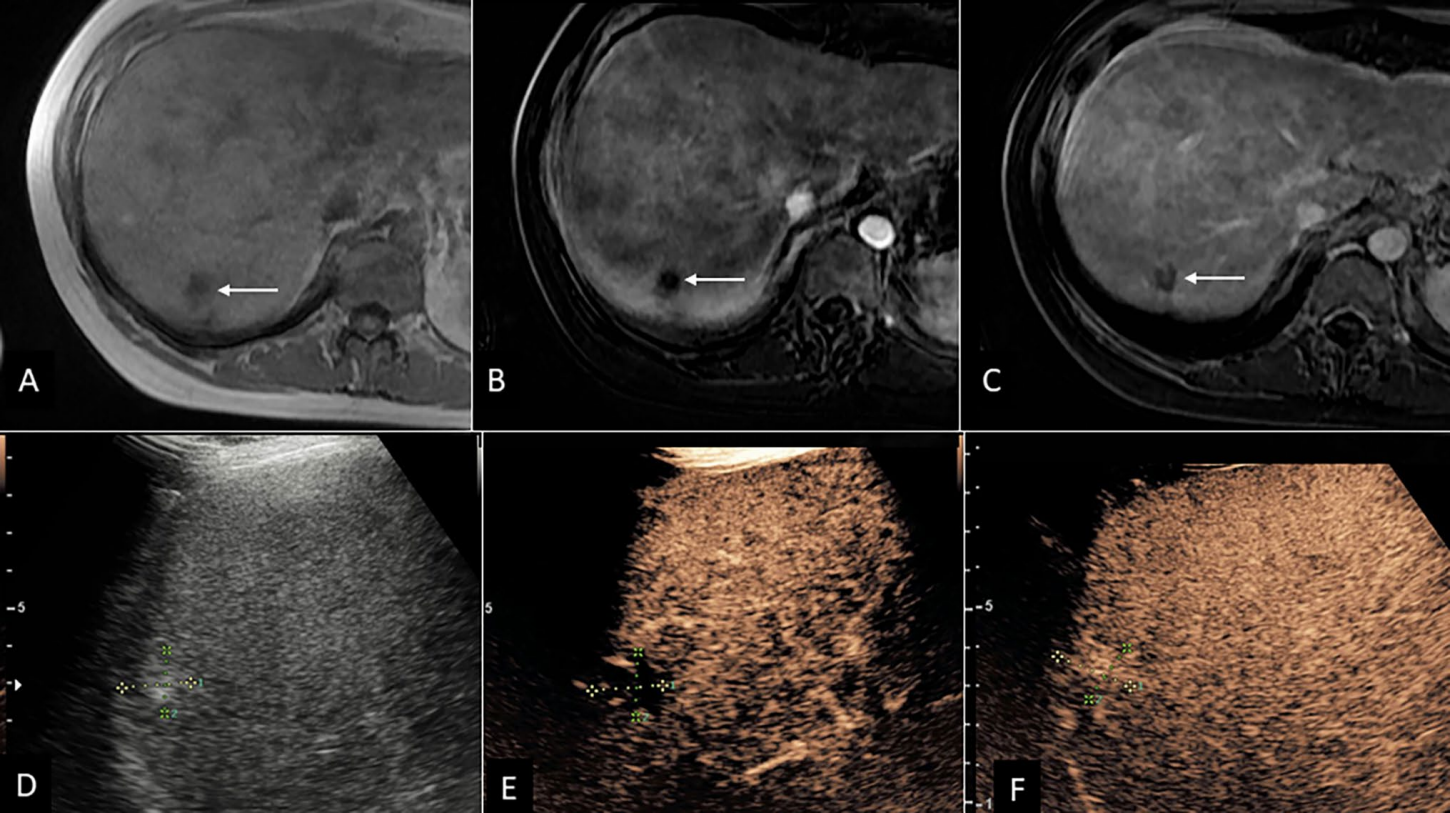
Hemangioma with MRI LR-3 categorization. **A** Pre-contrast MR image demonstrating small hypo-intense observation in the right hepatic dome (arrow). Arterial phase image which is degraded by motion artifact (**B**) demonstrated questionable arterial hypo enhancement in the parts of the observation, with hypoenhancement on late phase image (**C**), resulting in LR-3 categorization. **D** Grayscale ultrasound image showing hyperechoic liver nodule measuring 1.6 cm marked by calipers with peripheral interrupted APHE (**E**). CEUS representative image at 1 min (**F**) persistent hyperenhancement—typical appearance of hemangioma (LR-1)

**Fig. 2:**
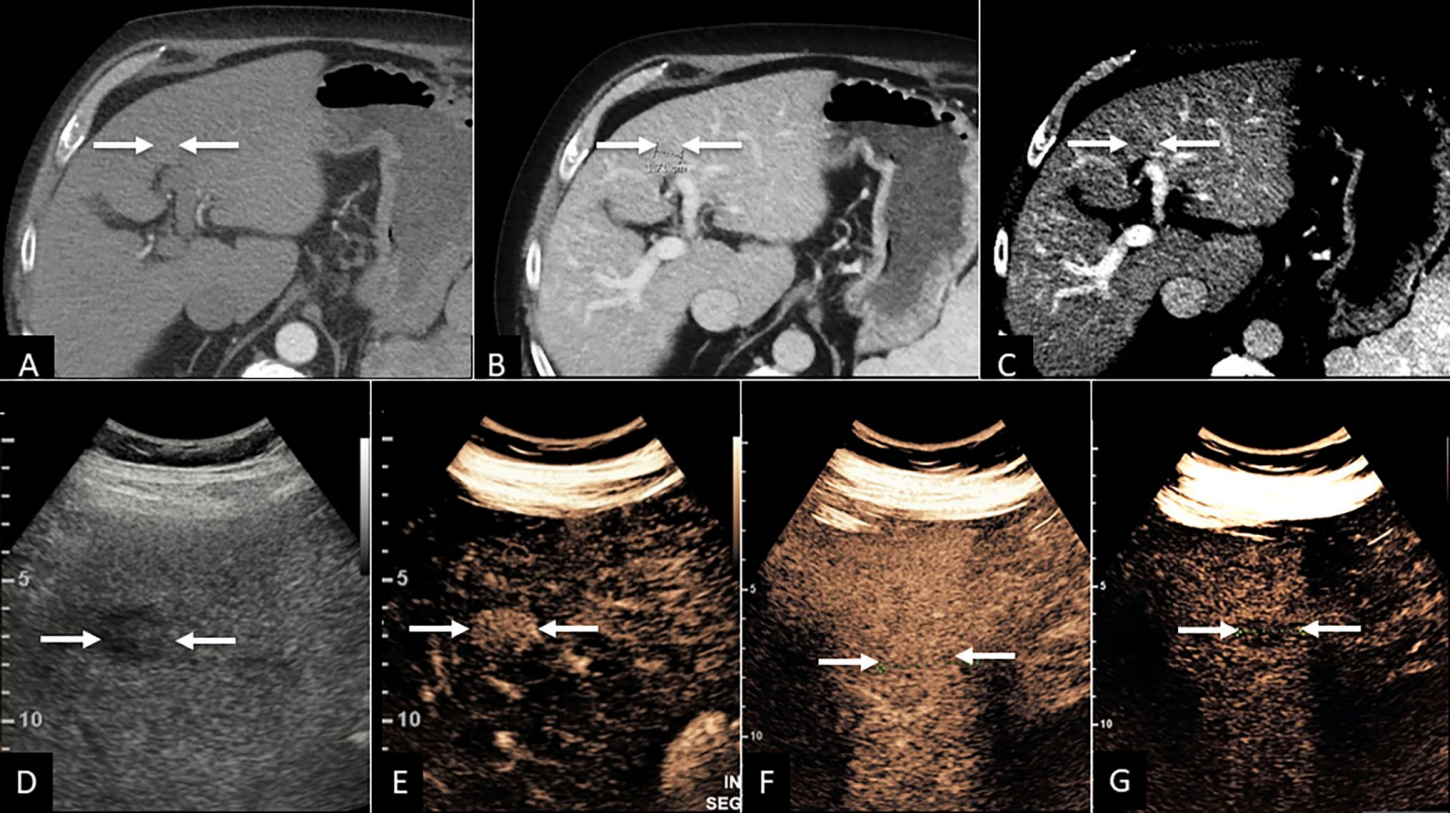
HCC with CT LR-3 categorization. **A** Arterial phase CT image demonstrating small nodule with no APHE (arrows). Later phase images (**B**, **C**) demonstrated barely perceptible washout. **D** Grayscale ultrasound image demonstrating hypoechoic nodule in a fatty liver measuring 2.5 cm with clear APHE (**E**). CEUS representative image at 1 min (**F**) showing no early washout. **G** CEUS representative image at 3 min demonstrating late and mild washout—typical appearance of HCC (LR-5), confirmed on biopsy

**Fig. 3:**
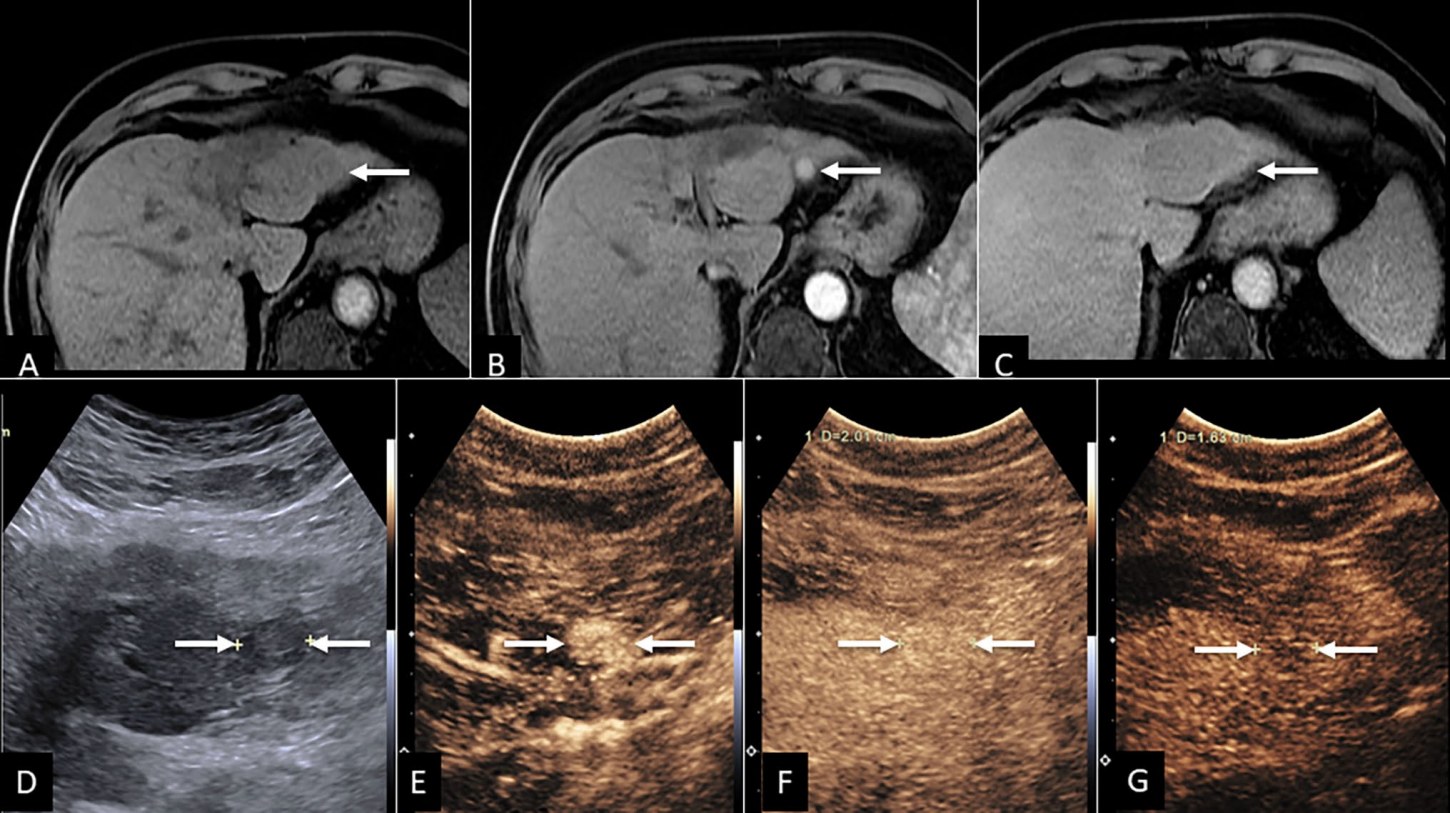
HCC with MRI LR-3 categorization. **A** Pre-contrast MR image demonstrating small iso-intense observation (arrow). Arterial phase image (**B**) demonstrated APHE without washout on late phase image (**C**). **D** Grayscale ultrasound image showing a small exophytic nodule measuring 1.5 cm with clear APHE (**E**). CEUS representative image at 1 min (**F**) showing no early washout. **G** CEUS representative image at 3 min demonstrating late and mild washout—typical appearance of HCC (LR-5), confirmed on explant histology

Of 54 CT/MRI LR-3 observations, CEUS categorized 13.0% (7/54) observations as non-malignant (1 LR-1 and 6 LR-2), all of which were confirmed non-malignant by reference standard, resulting in a CEUS NPV of 100% for HCC in this group (Fig. 1). An additional 13.0% (7/54) CT/MRI LR-3 observations were categorized on CEUS as LR-5, all of which were confirmed to be HCC by reference standard, resulting in a CEUS PPV of 100% for HCC in this group. Examples of CT/MRI LR-3 recategorization by CEUS to LR-5 are provided in Figs. 2 and 3.

**Fig. 4:**
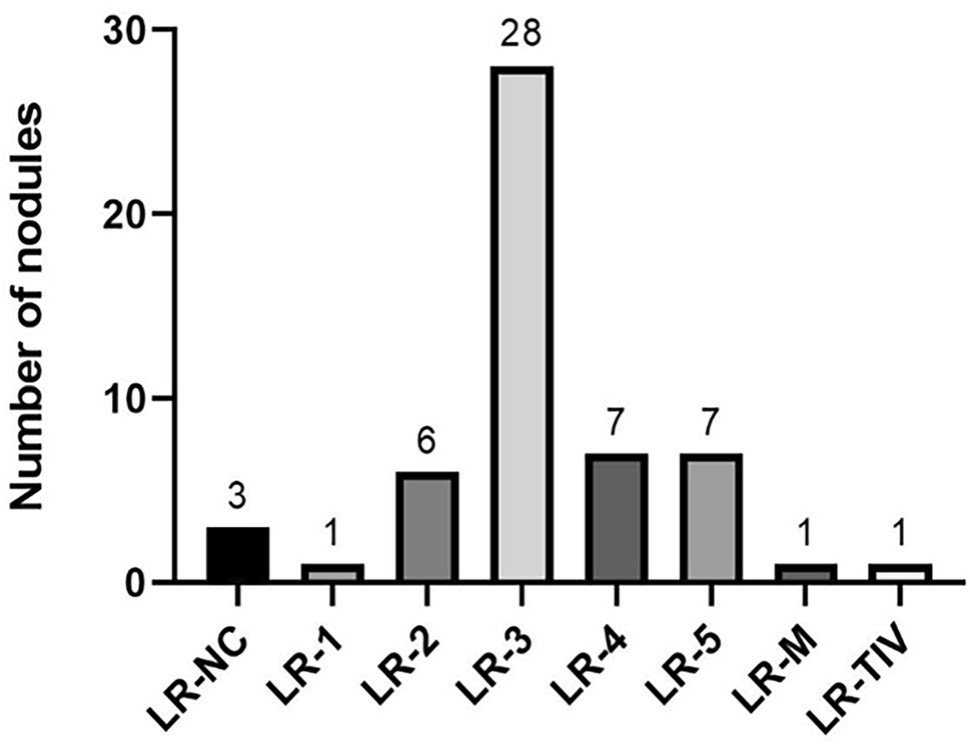
CEUS LI-RADS Results in Patients with LR-3 on CT/MRI

The remaining 40 (74.1%) CT/MRI LR-3 observations remained indeterminate after CEUS as follows: 51.9% (28/54) were categorized by CEUS as LR-3, 13.0% (7/54) as LR-4, 2% (1/54) as LR-M, 2% (1/54) LR-TIV, and 5.5% (3/54) as LR-NC (Fig. 4).

### Observations categorized as LR-NC on CT/MRI

**Fig. 5:**
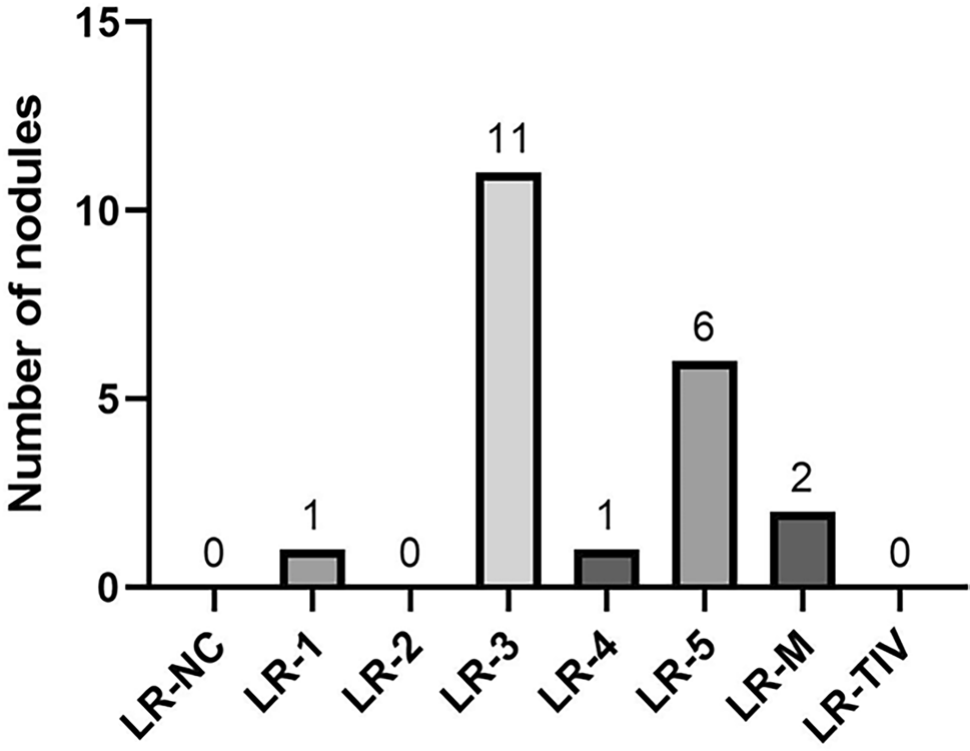
CEUS LI-RADS results in patients with LR-NC on CT/MRI

There were a total of 21 observations categorized as LR-NC on CT/MRI, 52.4% (11/21) were confirmed HCC, 42.9% non-malignant (9/21), and 4.8% non-hepatocellular malignancy (1/21). The final diagnosis reference standard was based on follow-up CT/MRI (66.7%, 14/21), percutaneous biopsy (28.6%, 6/21), or liver explant histology (4.8%, 1/21) (see Fig. 5).

**Fig. 6:**
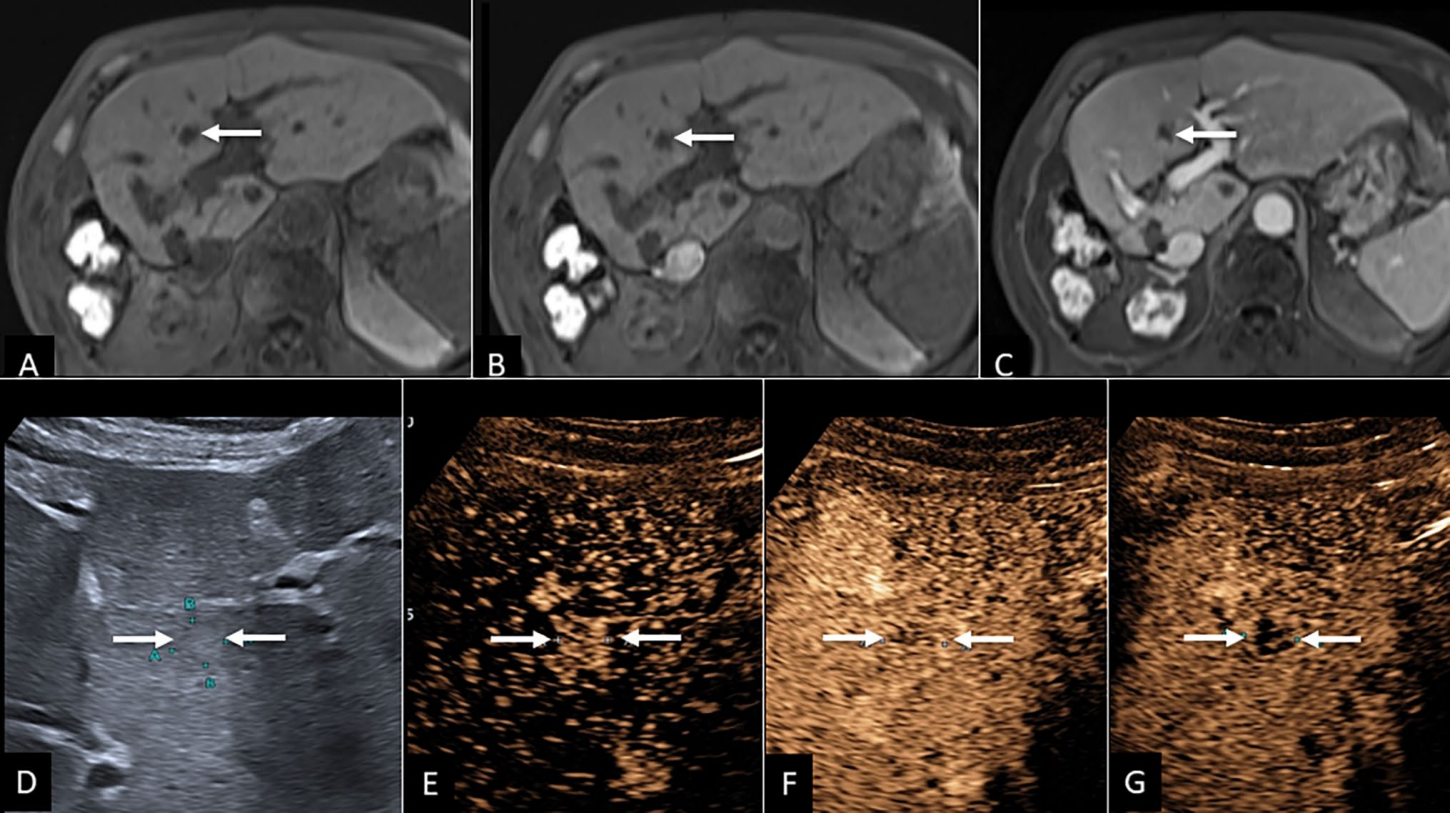
HCC with MRI LR-NC categorization. **A** Pre-contrast MR image demonstrating small hypo-intense observation (arrow). Arterial phase image (**B**) demonstrated no enhancement, likely due to mistiming of arterial phase, with persistent hypointensity on late phase image (**C**). **D** Grayscale ultrasound image showing small nodule measuring 1.1 cm with clear APHE (**E**). CEUS representative image at 1 min (**F**) showing no early washout. **G** CEUS representative image at 3 min demonstrating late and mild washout—typical appearance of HCC (LR-5), confirmed on follow-up MRI

Of 21 CT/MRI LR-NC observations, CEUS categorized one observation (4.8%, 1/21) as definitely benign (LR-1), confirmed as non-malignant by reference standard, resulting in a CEUS NPV of 100% for HCC in this group. An additional 38.9% (6/21) of CT/MRI LR-NC observations were categorized on CEUS as LR-5, all of which were confirmed HCC by reference standard, resulting in a CEUS PPV of 100% for HCC in this group. Example of CT/MRI LR-NC observation recategorization by CEUS to LR-5 is provided in Fig. 6.

The remaining 66.7% (14/21) CT/MRI LR-NC observations remained indeterminate after CEUS as follows: 52.4% (11/21) were categorized by CEUS as LR-3, 4.8% (1/21) as LR-4, and 9.5% (2/21) as LR-M (Fig. 4). Both observations categorized as LR-M on CEUS were confirmed malignant by reference standard (one lesion was confirmed HCC and another one as colon cancer metastasis). On note, none of the observations categorized as LR-NC on CT/MRI had the same LR-NC categorization on CEUS.

### Clinical impact of CEUS on CT/MRI LR-3 and LR-NC management recommendations

*In patients with CT/MRI LR-3 observations,* CEUS resulted in management recommendation changes in 29.6% (16/54) of observations and in 93.8% (15/16) of the re-categorized observations the recommendations were correct. A total of 25.9% (14/54) of observations were correctly categorized as non-malignant or definitely HCC, removing the need for follow-up imaging. One CEUS LR-TIV observation was referred to biopsy and confirmed as HCC, resulting in substantial change in patient management. Another observation was categorized as LR-M, also prompting recommendations for biopsy. The biopsy was not performed; the observation was downgraded to LR-2 on follow-up MRI and considered non-malignant.

*In patients with CT/MRI LR-NC observations,* CEUS resulted in management recommendation changes in 42.9% (9/21) of observations and in 100% (9/9) of the re-categorized observations the new management recommendations were correct. A total of 33.3% (7/21) of observations were correctly categorized as non-malignant or definitely HCC, alleviating the need for follow-up imaging. Another 9.5% (2/21) observations were categorized as LR-M prompting recommendation for tissue biopsy and both of these observations were confirmed malignant on tissue histology.

*If analyzed as a group including both CT/MRI LR-3 and LR-NC observations,* CEUS resulted in management recommendation changes in 33.3% (25/75) of observations and in all but one (96.0%, 24/25) of the re-categorized observations the new management recommendations were correct. A total of 28.0% (21/75) observations were correctly categorized as non-malignant or definitely HCC with 100% accuracy, alleviating the need for follow-up imaging. A single observation that was re-categorized as CEUS LR-TIV prompted recommendations for biopsy, confirming the tumor thrombus. Another 4% (3/75) of observations were re-categorized on CEUS as LR-M, also prompting recommendations for biopsy, which was correct in 66.7% (2/3) of the cases.

## Discussion

This multi-national study shows a relevant diagnostic benefit for the use of CEUS in a series of prospectively collected patients at risk for HCC, with liver observations categorized as indeterminate on CT or MRI. In nearly a third of cases originally deemed indeterminate on CT/MRI, CEUS achieved a definitive diagnosis with 100% PPV for CEUS LR-5 and LR-TIV and 100% NPV for CEUS LR-1 and LR-2.

Prior work attempting to resolve indeterminate liver findings focused primarily on repeating the CT/MRI, obtaining tissue sampling, or performing active surveillance. Abd Alkhalik Basha et al. conducted a study evaluating the diagnostic efficacy of LI-RADS with CT, categorizing small nodules detected in the cirrhotic liver at screening US. The study comprised 55 nodules, 14 of which were categorized as LR-3. Further investigation indicated that 7 of those nodules were finally confirmed as HCC, while the remaining lesions were benign [13]. Choi Se Jin et al. analyzed changes in size thresholds in CT/MRI according to LI-RADS v.2018 categorization and found 57 HCCs that were previously categorized as LR-3 (*n* = 3), LR-4 (*n* = 17), LR-5 (*n* = 31), and LR-M (*n* = 6). However, after the changes were implemented, it showed that the threshold had little impact on LI-RADS categorization for LR-5, although these changes showed that the observations that were initially categorized as LR-3 and LR-4 at CT/MRI could be re-categorized as LR-5 after 3–6 months imaging follow-up [14]. These findings agree with a 2018 study conducted by Mitchell et al. stating that the clinical outcome of nodules categorized as LR-3 with US evolves to HCC in a range of 6–69% [15]. Similarly, Darnell et al. evaluated 262 observations, of which 74 were categorized as LR-3 on MRI. Of these 74 LR-3 observations, with a median follow-up of 17 months, 51 nodules were diagnosed as HCC (68.9%), 2 were non-HCC malignant lesions (2.7%), and 21 remained as benign (28.37%) [16]. Shropshire et al. analyzed 141 lesions initially classified as LR-3 using the LI-RADS v2017 classification, with a mean follow-up imaging, either contrast-enhanced CT or MR, of 20.3 ± 13.4 months. From the initial classification, 40% (57/141) of the lesions remained as LR-3; 50% were downgraded to a benign classification (59 LR-1, 11 LR-2); 2% (3/141) were upgraded to LR-4; and 8% (11/141) was upgraded to LR-5 [17].

There are also clinical studies comparing inter-reader agreement and how their discrepancies can cause changes in management and outcomes. For instance, Yokoo et al. studied nodules categorized as LR-3, LR-4, LR-5, and LR-M and found that there was more discordance categorizing LR-3 and LR-4 observations than LR-5 observations and highlighted how this error may have affected management, including decisions regarding therapy and staging for organ transplantation [18]. In this study of 69 patients, 53 had discordant results, from which 30 had changes in clinical management. The reasons for discordance were detection (20/30), size (2/30), and LR category (8/30). In the detection discordance, the clinical change was type of follow-up imaging (between bi-annual screening US and shorter-term FU CT/MRI) (15/20), referral for biopsy (2/20), and eligibility for transplant (3/20). Discordance due to LR category consisted mainly in choosing imaging modality for follow-up (3/8), referral for biopsy (3/8), and changes between treatment modality (2/8). When the size classification was discordant the clinical changes made were between choosing locoregional vs systemic therapy and the eligibility of one patient to be considered for transplant.

Similarly, Smereka et al. studied LI-RADS in MRI, comparing the 2017 vs 2018 version between 3 radiologists. The combined data of all 3 radiologists showed that with the 2017 criteria, 42 out of 110 nodules (38.1%) categorized as LR-3 progressed to HCC, and while using the 2018 criteria, 144 out of 257 nodules (44.4%) categorized as LR-3 progressed to HCC. Additionally, the group showed that with the revised guidelines, several observations re-categorized as LR-3 had measurements between 10 and 19 mm [19].

Prior studies also compared the prognosis of LR-3 lesions identified by CEUS and CT/MRI. Zhou et al. conducted a meta-analysis comparing the proportion of HCC between the respective CEUS LI-RADS and CT/MRI LI-RADS categories and the proportion of HCC and non-HCC malignancies in each. They found that the proportion of HCC in CEUS LR-3 is lower than in CT/MRI (21% vs 35%). In addition, it outlined that there was more HCC categorized as LR-2, 3, and 4 in CT/MRI than in CEUS [20]. These data demonstrate the potential advantages of CEUS for indeterminate liver nodule categorization as both a primary and secondary diagnostic tool.

LR-NC category is assigned to those cases where there are technical or patient-related limitations that prevent the characterization of an observation, such as the complete lack of arterial phase images [21, 22]. Elsayes et al. recommended that nodules categorized as LR-NC may need repeat imaging tests in less than 3 months [23]. Kamath et al. reported one LR-NC case where the initial test was non-characterizable due to artifacts present on non-contrast images, and the inability to conclude the test with contrast. For this reason, the test modality was changed to multiphase CT, in which the observation was categorized as LR-5 [22].

Our study has several limitations, the most important of which is use of a composite imaging and histology reference standard. Ideally, HCC diagnosis should be established based on tissue sampling. However, the current clinical practice is based on imaging-based noninvasive diagnosis of HCC introduced by the Barcelona-2000 European Association for the Study of the Liver conference [2]. Therefore, obtaining histological diagnosis for every (or even most) HCC is no longer standard of care, making it practically impossible to use histology as reference in every case. Also, for this study, observations with persistent CT/MRI LR-3 categorization on 1-year follow-up imaging were considered non-malignant. However, since the growth rate of some well-differentiated HCC is relatively small, a 1-year follow-up may not be long enough to exclude malignancy. On the other hand, nodules followed for more than 1 year and initially categorized as non-malignant may eventually transform to malignancy. Another limitation is that similar to routine clinical practice, in most cases readers of CEUS were not blinded to the images/results of CT/MRI, since CT/MRI results commonly used to guide CEUS examinations. Although doing so might increase clinical applicability of our study findings. Also, despite the prospective multicenter design of our study, the relatively small sample size (75 liver lesions) of this sub-analysis may affect the generalizability and statistical power of the findings.

These findings have significant implications for patient management, and based on our data and the results of others, we believe that in patients at risk for HCC that CEUS should be recommended as an appropriate next step in management of focal liver observations with indeterminate CT/MRI categorization.

## Conclusion

CEUS is a safe and effective tool that well complements CT/MRI diagnosis of HCC. In our study, CEUS LI-RADS demonstrated high diagnostic benefit in liver observations with inconclusive or indeterminate categorization on CT/MRI. Therefore, CEUS should be strongly considered as an appropriate next step in management for focal liver observations with inconclusive or indeterminate CT/MRI categorization in patients at risk for HCC since it accelerates the diagnostic process in a considerable number of patients.

## Abbreviations

AASLD: American Association for the Study of Liver Diseases
ACR: American College of Radiology
CE-MRI: Contrast-enhanced magnetic resonance imaging
CEUS: Contrast-enhanced ultrasound
CT: Computed tomography
EASL: European Association for the Study of the Liver
ECOG: Eastern Cooperative Oncology Group
HCC: Hepatocellular carcinoma
LI-RADS: Liver imaging reporting and data system
LR: LI-RADS
MRI: Magnetic resonance imaging
NCCN: National comprehensive cancer network
NPV: Negative predictive value
NASH: Non-alcoholic Steatohepatitis
LR-NC: Non-characterizable lesions
OPTN: Organ procurement and transplantation network
PPV: Positive predictive value
SAS: Statistical analysis software
LR-TIV: Tumor in vein
US: Ultrasound
UNOS: United network for organ sharing
USA: United States of America

## References

[1] Candita G, Rossi S, Cwiklinska K, Fanni SC, Cioni D, Lencioni R, et al. Imaging Diagnosis of Hepatocellular Carcinoma: A State-of-the-Art Review. Diagn Basel Switz. 2023 Feb 8;13(4):625. 10.3390/diagnostics13040625 PMC9955560 36832113

[2] Singal AG, Llovet JM, Yarchoan M, Mehta N, Heimbach JK, Dawson LA, et al. AASLD Practice Guidance on prevention, diagnosis, and treatment of hepatocellular carcinoma. Hepatol Baltim Md. 2023 Dec 1;78(6):1922–65. 10.1097/HEP.0000000000000466 PMC10663390 37199193

[3] Chernyak V, Fowler KJ, Do RKG, Kamaya A, Kono Y, Tang A, et al. LI-RADS: Looking Back, Looking Forward. Radiology. 2023 Apr;307(1):e222801. 36853182 10.1148/radiol.222801 PMC10068888

[4] Benson AB, D’Angelica MI, Abbott DE, Anaya DA, Anders R, Are C, et al. Hepatobiliary Cancers, Version 2.2021, NCCN Clinical Practice Guidelines in Oncology. J Natl Compr Cancer Netw JNCCN. 2021 May 1;19(5):541–65. 10.6004/jnccn.2021.0022 34030131

[5] European Association for the Study of the Liver. Electronic address: easloffice@easloffice.eu, European Association for the Study of the Liver. EASL Clinical Practice Guidelines: Management of hepatocellular carcinoma. J Hepatol. 2018 Jul;69(1):182–236. 10.1016/j.jhep.2018.03.019 29628281

[6] Omata M, Cheng AL, Kokudo N, Kudo M, Lee JM, Jia J, et al. Asia-Pacific clinical practice guidelines on the management of hepatocellular carcinoma: a 2017 update. Hepatol Int. 2017 Jul;11(4):317–70. 28620797 10.1007/s12072-017-9799-9 PMC5491694

[7] Burak KW, Sherman M. Hepatocellular carcinoma: Consensus, controversies and future directions. A report from the Canadian Association for the Study of the Liver Hepatocellular Carcinoma Meeting. Can J Gastroenterol Hepatol. 2015 May;29(4):178–84. 10.1155/2015/824263 PMC4444026 25965437

[8] Lee JM, Park JW, Choi BI. 2014 KLCSG-NCC Korea Practice Guidelines for the management of hepatocellular carcinoma: HCC diagnostic algorithm. Dig Dis Basel Switz. 2014;32(6):764–77. 10.1159/000368020 25376295

[9] Kudo M, Matsui O, Izumi N, Iijima H, Kadoya M, Imai Y, et al. JSH Consensus-Based Clinical Practice Guidelines for the Management of Hepatocellular Carcinoma: 2014 Update by the Liver Cancer Study Group of Japan. Liver Cancer. 2014 Oct;3(3–4):458–68. 26280007 10.1159/000343875 PMC4531423

[10] Jang HJ, Kim TK, Burns PN, Wilson SR. CEUS: An essential component in a multimodality approach to small nodules in patients at high-risk for hepatocellular carcinoma. Eur J Radiol. 2015 Sep;84(9):1623–35. 26092406 10.1016/j.ejrad.2015.05.020

[11] Lyshchik A, Wessner CE, Bradigan K, Eisenbrey JR, Forsberg F, Yi M, Keith SW, Kono Y, Wilson SR, Medellin A, Rodgers SK, Planz V, Kamaya A, Finch L, Fetzer DT, Berzigotti A, Sidhu PS, Piscaglia F; CEUS LI-RADS Trial Group. Contrast-enhanced ultrasound liver imaging reporting and data system: clinical validation in a prospective multinational study in North America and Europe. Hepatology. 2024 Feb 1;79(2):380–391. doi: 10.1097/HEP.0000000000000558. Epub 2023 Aug 8. PMID: 37548928.; 10.1097/HEP.0000000000000558 PMC11810132 37548928

[12] Terzi E, Giamperoli A, Iavarone M, Leoni S, De Bonis L, Granito A, et al. Prognosis of Single Early-Stage Hepatocellular Carcinoma (HCC) with CEUS Inconclusive Imaging (LI-RADS LR-3 and LR-4) Is No Better than Typical HCC (LR-5). Cancers. 2022 Jan 11;14(2):336. 35053498 10.3390/cancers14020336 PMC8773738

[13] Abd Alkhalik Basha M, Abd El Aziz El Sammak D, El Sammak AA. Diagnostic efficacy of the Liver Imaging-Reporting and Data System (LI-RADS) with CT imaging in categorising small nodules (10–20 mm) detected in the cirrhotic liver at screening ultrasound. Clin Radiol. 2017 Oct;72(10):901.e1–901.e11. 10.1016/j.crad.2017.05.019 28673446

[14] Choi SJ, Choi SH, Kim DW, Kwag M, Byun JH, Won HJ, et al. Value of threshold growth as a major diagnostic feature of hepatocellular carcinoma in LI-RADS. J Hepatol. 2023 Mar;78(3):596–603. 36402451 10.1016/j.jhep.2022.11.006

[15] Mitchell DG, Bashir MR, Sirlin CB. Management implications and outcomes of LI-RADS-2, -3, -4, and -M category observations. Abdom Radiol N Y. 2018 Jan;43(1):143–8. 10.1007/s00261-017-1251-z 28779335

[16] Darnell A, Rimola J, Belmonte E, Ripoll E, Garcia-Criado Á, Caparroz C, et al. Evaluation of LI-RADS 3 category by magnetic resonance in US-detected nodules ≤ 2 cm in cirrhotic patients. Eur Radiol. 2021 Jul;31(7):4794–803. 33409789 10.1007/s00330-020-07457-6

[17] Shropshire E, Mamidipalli A, Wolfson T, Allen BC, Jaffe TA, Igarashi S, et al. LI-RADS ancillary feature prediction of longitudinal category changes in LR-3 observations: an exploratory study. Abdom Radiol. 2020 Oct;45(10):3092–102. 10.1007/s00261-020-02429-2 32052132

[18] Yokoo T, Singal AG, Diaz de Leon A, Ananthakrishnan L, Fetzer DT, Pedrosa I, et al. Prevalence and clinical significance of discordant LI-RADS® observations on multiphase contrast-enhanced MRI in patients with cirrhosis. Abdom Radiol N Y. 2020 Jan;45(1):177–87. 10.1007/s00261-019-02133-w 31342103

[19] Smereka P, Doshi AM, Lavelle LP, Shanbhogue K. New Arterial Phase Enhancing Nodules on MRI of Cirrhotic Liver: Risk of Progression to Hepatocellular Carcinoma and Implications for LI-RADS Classification. AJR Am J Roentgenol. 2020 Aug;215(2):382–9. 32432909 10.2214/AJR.19.22033

[20] Zhou Y, Qin Z, Ding J, Zhao L, Chen Y, Wang F, et al. Risk Stratification and Distribution of Hepatocellular Carcinomas in CEUS and CT/MRI LI-RADS: A Meta-Analysis. Front Oncol. 2022;12:873913. 35425706 10.3389/fonc.2022.873913 PMC9001845

[21] Santillan C, Chernyak V, Sirlin C. LI-RADS categories: concepts, definitions, and criteria. Abdom Radiol N Y. 2018 Jan;43(1):101–10. 10.1007/s00261-017-1334-x 29038857

[22] Kamath A, Roudenko A, Hecht E, Sirlin C, Chernyak V, Fowler K, et al. CT/MR LI-RADS 2018: clinical implications and management recommendations. Abdom Radiol N Y. 2019 Apr;44(4):1306–22. 10.1007/s00261-018-1868-6 30671612

[23] Elsayes KM, Kielar AZ, Chernyak V, Morshid A, Furlan A, Masch WR, et al. LI-RADS: a conceptual and historical review from its beginning to its recent integration into AASLD clinical practice guidance. J Hepatocell Carcinoma. 2019;6:49–69. 30788336 10.2147/JHC.S186239 PMC6368120

